# Neural correlates of a load-dependent decline in visual working memory

**DOI:** 10.1093/texcom/tgac015

**Published:** 2022-04-09

**Authors:** Yaju Li, Yasuki Noguchi

**Affiliations:** Department of Psychology, Graduate School of Humanities, Kobe University, 1-1 Rokkodai-cho, Nada, Kobe 657-8501, Japan; Department of Psychology, Graduate School of Humanities, Kobe University, 1-1 Rokkodai-cho, Nada, Kobe 657-8501, Japan

**Keywords:** alpha frequency, beta frequency, change detection, electroencephalography, memory degradation

## Abstract

Previous studies have shown that a rate of temporal decline in visual working memory (vWM) highly depends on a number of memory items. When people retain the information of many (≥ 4) stimuli simultaneously, their memory representations are fragile and rapidly degrade within 2–3 s after an offset (called the “competition” among memory items). When a memory load is low (1 or 2 items), in contrast, the fidelity of vWM is preserved for a longer time because focused attention to the small number of items prevents the temporal degradation. In the present study, we explored neural correlates of this load-dependent decline of vWM in the human brain. Using electroencephalography and a classical change-detection task, we recorded neural measures of vWM that have been reported previously, such as the contralateral delay activity (CDA) and a suppression of alpha power (8–12 Hz). Results indicated that the load-dependent decline of vWM was more clearly reflected in the change in power and speed of alpha/beta rhythm than CDA, suggesting a close relationship of those signals to an attention-based preservation of WM fidelity.

## Introduction

Although visual working memory (vWM) plays a critical role in a variety of cognitive tasks, its neural correlates remain unclear and a matter of debate (Baddeley 2012; Luck and Vogel 2013; Ma et al. 2014; D'Esposito and Postle 2015; Eriksson et al. 2015; Stokes 2015; Kaminski et al. 2017; Leavitt et al. 2017; Jacob et al. 2018). Some researchers indicated an importance of persistent neuronal activity in the frontoparietal region for maintenance of WM (Constantinidis et al. 2018), whereas others reported a close relationship of WM with oscillatory signals (Roux and Uhlhaas 2014; Johnson et al. 2017; van Ede 2018), such as theta (4–8 Hz) and gamma (>30 Hz) rhythms (Lundqvist et al. 2018). In humans, a representative neural measure of vWM is the contralateral delay activity (CDA), an event-related potential (ERP) observed in the parieto-occipital cortex (Vogel and Machizawa 2004; Wang et al. 2019). More recent studies reported a role of cross-frequency coupling (Canolty et al. 2006; Sauseng et al. 2009; Axmacher et al. 2010; Lisman and Jensen 2013; Reinhart and Nguyen 2019), such as phase-amplitude coupling between theta and gamma bands. Other candidates include a change in power of alpha (Jensen et al. 2002; Bonnefond and Jensen 2012; Wianda and Ross 2019) and beta (Palva et al. 2011; Erickson et al. 2017; van Ede et al. 2017) rhythms, as well as change in their oscillation frequency (Moran et al. 2010; Cohen 2011; Babu Henry Samuel et al. 2018; Noguchi and Kakigi 2020).

Most previous studies have investigated neural activity during a retention interval (maintenance) of WM task. In the present study, we approached this issue by focusing on a decline (rather than the maintenance) of vWM. Recent studies showed that a rate of temporal decline in WM highly depends on a number of memory items (Lewis-Peacock and Norman 2014; Pertzov et al. 2017; Panichello et al. 2019). When people memorized 1 or 2 visual items simultaneously presented, they can reproduce the items in fine detail after a retention period of 2–3 s. When a number of memory items was large (≥ 4), however, their memory representations rapidly degraded within a few seconds. This decline in the high-load conditions is thought to reflect a competition between items that accelerated degradation of memory representations (Pertzov et al. 2017).

In Experiment 1 of the present study, we replicated this finding using a traditional paradigm of WM in which participants memorized colors of multiple visual items (Vogel and Machizawa 2004). We then recorded human electroencephalography (EEG) signals and tracked changes in vWM measures (e.g. CDA and alpha amplitude) over time (Exp. 2). We hypothesized that neural measures reflecting a fidelity of WM would show the load-dependent decline described above. Specifically, memory-related signal in those measures would show a substantial decline from the early to late periods of a retention interval in high-load conditions, while it would be preserved in low-load conditions.

## Materials and methods

### Participants

Fourteen healthy subjects (8 females, age: 20–23) participated in Experiment 1 (behavioral study), and 42 healthy subjects participated in Experiment 2 (EEG study). In Experiment 2, we excluded the data of 5 subjects from analysis due to a technical problem (disconnection of a trigger line, *n* = 3), excessive noise in EEG waveforms (*n* = 1), and a poor behavioral performance (memory capacity *K* < 0 in R6 trials, see below, *n* = 1), leaving 37 participants in the final dataset (20 females, age range 19–22). All participants had normal or corrected-to-normal visual acuity and normal color vision. After the nature of the study had been explained, we received informed consent from each participant. All experiments were conducted in accordance with regulations and guidelines approved by the Ethics Committee of Kobe University, Hyogo, Japan.

### Stimuli and task (Exp. 1)

**Fig. 1:**
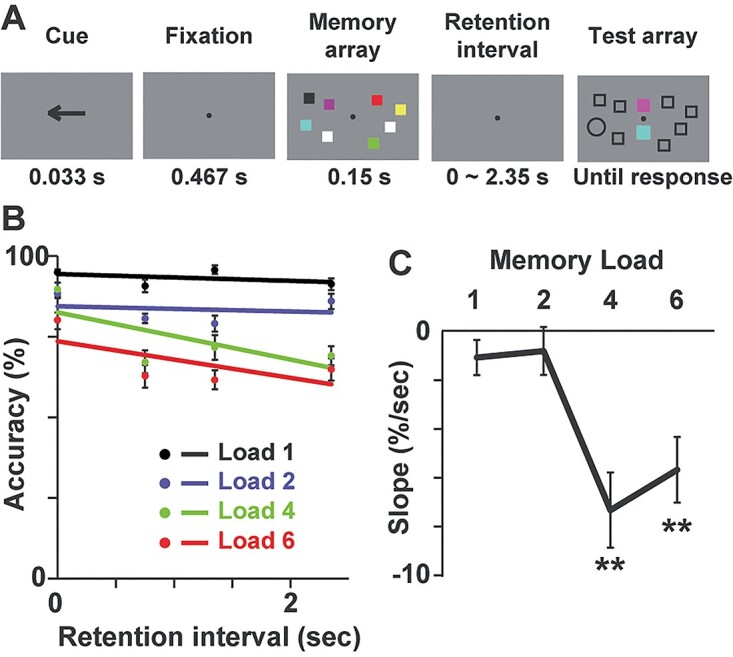
Experiment 1 (behavioral study). **A**) Sequence of 1 trial. Participants memorized items in a hemifield (left or right) indicated by a cue (arrow). After a random delay of 0–2,350 ms, they reported a color of an item that had been presented at a location indicated by a blank circle. Specifically, they chose one of 2 colors (options) in the central field of the test array (chance level: 50%). **B**) Task accuracy as a function of a retention interval. Dots and error bars in each color (black: load 1, blue: load 2, green: load 4, red: load 6) show mean ± SE across 14 participants with a solid line indicating a linear regression of those data. **C**) Slopes of the regression line. Negative slopes observed in load 4 and load 6 showed a temporal decay of vWM in those conditions. ^*^^*^P < 0.01, 1-group *t*-test corrected with the Bonferroni method.

In Experiment 1, we aimed to replicate the load-dependent decline of vWM (Lewis-Peacock and Norman 2014; Pertzov et al. 2017; Panichello et al. 2019) with a classical paradigm in which participants retained color-position associations of stimuli (squares) in a left or right visual field. All stimuli were presented using the Matlab Psychophysics Toolbox (Brainard 1997; Pelli 1997) and a CRT monitor at a refresh rate of 60 Hz. Each trial was preceded by a black fixation point (0.18 × 0.18 deg.) over a gray background for 800 ms. Next, a cue stimulus (an arrow pointing leftward or rightward, 1.34 deg.) appeared over the central field for 33 ms (Fig. 1A) to direct attention of participants. After another fixation period of 467 ms, participants viewed a bilateral array of colored squares for 150 ms (memory array). The array had an equal number of squares in left and right visual fields (1, 2, 4, or 6 per hemifield, variable across trials). For each of basic 9 colors (black, white, red, green, blue, yellow, magenta, cyan, and orange) used in a previous study (Vogel and Machizawa 2004), we generated 2 variants with different hues; for example, 2 equi-luminant colors, greenish blue and reddish blue, were generated as variants of blue. The color of each memory item in Experiment 1 was randomly chosen from those 18 colors (9 basic colors × 2 variants). Their locations were also randomly selected over 2 invisible rectangular regions of 3.89 deg (horizontal) × 6.81 deg (vertical) in both hemifields (eccentricity: 3.4 deg.), with the constraint that a minimum distance between squares was 1.94 deg.

After a retention period of 0, 750, 1,350, or 2,350 ms (variable across trials), another array consisting of a blank circle and squares (test array) was displayed until participants pressed any key. Positions of the blank items were identical to those in memory-array items, with the blank circle always located in the cued hemifield. Participants were asked to move their attention following the cue and to report a color of a memory item that had been presented at the position of the blank circle. Specifically, we showed a pair of color variants (e.g. greenish blue and reddish blue) in the test array, one just above and the other below a fixation point (options). Participants answered the color of the memory item at the circle position by choosing 1 of the 2 options (chance level: 50%). They were also informed that the blank circle always appeared in the cued hemifield. Because an exact position of the blank circle had been unknown till the test array in load-2, load-4 and load-6 trials, participants had to memorize all items in the cued hemifield during the retention period.

A combination of memory loads (1/2/4/6) and retention intervals (0, 750, 1,350, 2,350 ms) produced 16 types of trials. Each experiment consisted of 432 trials (72 trials × 6 sessions) in which those 16 conditions were randomly intermixed. A rate of memory decline was measured by estimating a slope of changes in task accuracy as a function of retention period.

### Stimuli and task (Exp. 2)

**Fig. 2:**
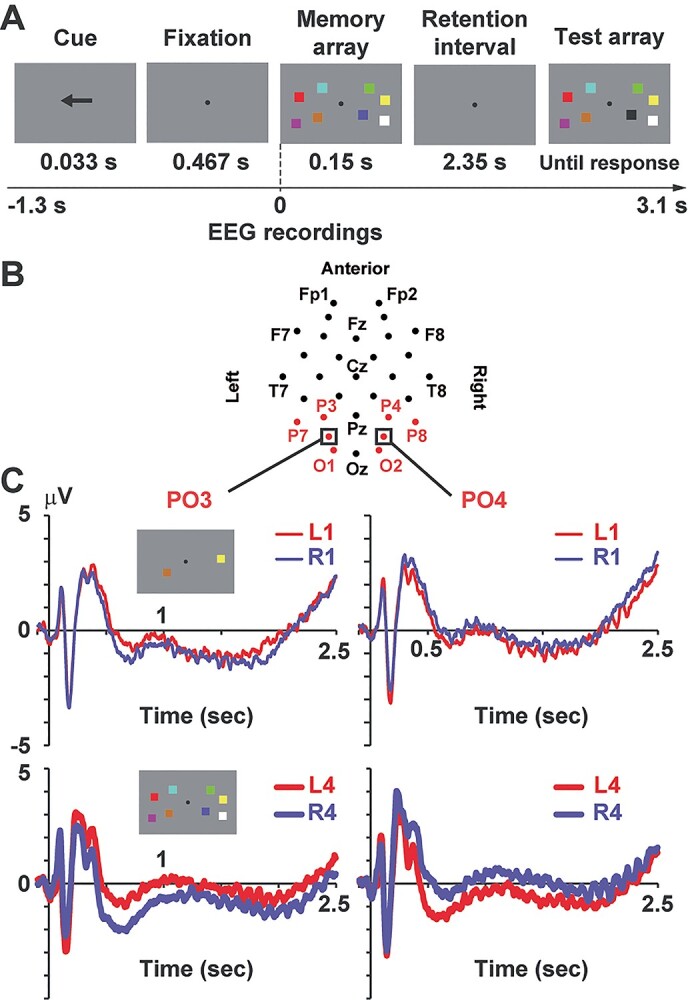
Experiment 2 (EEG study). **A**) the cued change-detection task. After a cue to direct attention of participants, memory and test arrays were sequentially presented with a retention interval between them. The 2 arrays were identical (no-change trials) or differed by 1 color (change trials). Participants moved their attention to a cued hemifield and answered whether the 2 arrays were the same (“no-change” response) or not (“change” response). **B**) Two-dimensional array of 32 EEG electrodes. We chose 8 electrodes over the parieto-occipital regions (shown in red) as SOIs, based on previous studies (see text). (**C**) ERP averaged across all participants. Waveforms at the left parieto-occipital sensor (PO3) showed a negative ERP component (CDA) when participants memorized items (squares) in right visual field (R1 and R4 trials, blue lines) compared with when they memorized items in left visual field (L1 and L4, red lines). This pattern was reversed in the right hemisphere (PO4). The CDA was more clearly seen when a number of memory items (load) was 4 (L4 vs. R4, lower panels) than 1 (L1 vs. R1, upper panels).

In Experiment 2, we investigated two representative EEG measures of vWM (CDA and alpha amplitude) and tracked their changes over time. A sequence of 1 trial is shown in Fig. 2A. After a cue of 33 ms, memory array was presented for 150 ms containing an equal number of items in left and right visual fields (1–6). Participants were asked to memorize the squares in a hemifield (left or right) indicated by the cue. After a retention period of 2350 ms (fixation screen), we tested their memory by presenting the second array (test array) that either was identical to the memory array (no-change trials) or differed by 1 color (change trials). Participants judged whether memory items (squares) in the cued hemifield were the same (“no-change” response) or not (“change” response) between the two arrays, ignoring all items in uncued hemifield (cued change-detection task).

There were 8 types of trials produced by a combination of cued hemifield (left/right) and memory array size (WM load, 1/2/4/6). Trials with leftward cue and 1 memory item were named as L1, whereas trials with rightward cue and 6 memory items were called as R6. The 9th condition (catch trials) was also included to check whether participants moved their attention to the cued hemifield. The memory and test arrays in those catch trials had 2 squares in each hemifield. While the color change usually occurred in the cued hemifield, it took place in the uncued hemifield in the catch trials (hence, participants should press “no-change” button). A high rate of reporting “change” in the catch trials would indicate that a participant did not allocate his/her attention following the cue.

An experimental session consisted of 100 trials in which 4 catch trials were randomly intermixed with the 96 trials of the 8 conditions (L1–R6, 12 trials for each). A ratio of the change: no-change trials in L1–R6 was 1:1. Each participant underwent 5 sessions.

We analyzed behavioral data by computing a hit rate (percentage of reporting “change” in the change trials) and false-alarm (FA) rate (percentage of reporting “change” in the no-change trials) for each memory load. Memory capacity *K* was also estimated using the hit and FA rates based on the formula below (Pashler 1988; Cowan 2001).

$$ K=S\times \left(\mathrm{Hit}-\mathrm{FA}\right) $$

where S indicate the size of memory array (1, 2, 4, or 6).

### E‌EG measurements and preprocessing

Neural activity was recorded with the ActiveTwo system by Biosemi (Amsterdam, Netherlands). We measured EEG signals at 32 points over the scalp (FP1, FP2, AF3, AF4, F7, F3, Fz, F4, F8, FC5, FC1, FC2, FC6, T7, C3, Cz, C4, T8, CP5, CP1, CP2, CP6, P7, P3, Pz, P4, P8, PO3, PO4, O1, Oz, and O2, Fig. 2B) with a sampling rate of 2,048 Hz and an analog low-pass filter of 417 Hz. The Brainstorm toolbox for MATLAB (Tadel et al. 2011) was used to perform the preprocessing of EEG data. We first applied a band-pass filter of 0.5–200 Hz to eliminate low- and high-frequency noises. All data were referenced with an average potential over the 32 electrodes. We then segmented EEG waveforms into each trial (epoch range: −1,300 to 3,100 ms relative to an onset of the memory array) and classified them into the 9 conditions. Waveforms with a max–min amplitude larger than 150 μV at −700 to 2,500 ms were excluded from analyses. Across-trial averaging was performed to identify the CDA (Fig. 2C).

### Analyses of speed and amplitude of oscillatory signals

As a main oscillatory signal related to vWM, we analyzed change in an amplitude of alpha rhythm (8–12 Hz) contralateral to a cued hemifield (Jensen et al. 2002; Hakim et al. 2020; Wang et al. 2021). First, we extracted the alpha rhythm in EEG waveforms using a band-pass filter of 8–12 Hz. An envelope of the filtered waveform was identified with the Hilbert transformation. An across-trial average of the envelope reflected changes in alpha amplitude over time.

### Statistical procedures

**Fig. 3:**
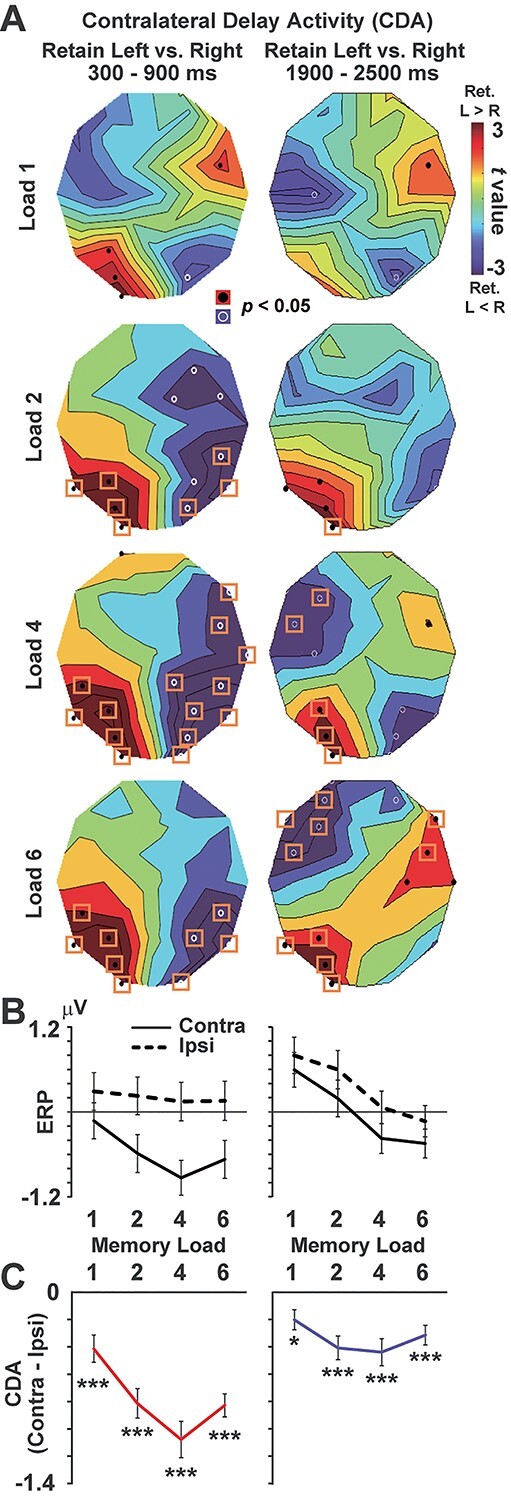
Temporal decline of the CDA. **A**) The *t*-map between Retain-Left trials (L1, L2, L4, and L6) and Retain-Right trials (R1, R2, R4, and R6). Top panels show a comparison of L1 versus R1 (load 1), whereas L6 and R6 were compared in the bottom panels (load 6). Results in early (300–900 ms) and late (1900–2500 ms) periods of retention interval are displayed in left and right panels, respectively. Black dots and white circles show sensor positions showing a significant (*P* < 0.05, uncorrected) difference. Orange rectangles denote a significant difference after a correction of multiple comparisons (see texts). **B**) Mean ERP averaged across the 8 SOIs (Fig. 2B). Solid/dotted lines show the data when participants memorized items in a visual field contralateral/ipsilateral to a sensor position. **C**) Difference between contralateral and ipsilateral conditions (CDA). ^*^P < 0.05, ^*^^*^^*^P < 0.001, 1-group *t*-test corrected with the Bonferroni method. All error bars denote SE across participants.

Neural responses related to vWM were identified by the difference between Retain-Left trials (L1, L2, L4, and L6) and Retain-Right trials (R1, R2, R4, and R6). For example, ERP waveforms in the left hemisphere became more negative when items in right visual field was retained (Retain-Left > Retain-Right, Fig. 2C), whereas this was reversed in the right hemisphere (Retain-Left < Retain-Right). Contrasting Retain-Left and Retain-Right trials thus showed hemisphere-specific neural responses related to WM maintenance (CDA, *t*-maps in Fig. 3A). The memory-related responses should be larger in high-load trials (L4 vs. R4 and L6 vs. R6) than low-load trials (L1 vs. R1 and L2 vs. R2) at least at 300–900 ms (load effect) (Vogel and Machizawa 2004). Because a paired *t*-test (Retain-Left vs. Retain-Right, *n* = 37) was repeated for 32 sensor positions, we resolved an issue of multiple comparisons by false discovery rate approach, adjusting a significance threshold with the Benjamini–Hochberg correction (Benjamini and Hochberg 1995). Sensors showing a significant difference after the correction are shown by orange rectangles in Fig. 3A.

**Fig. 4:**
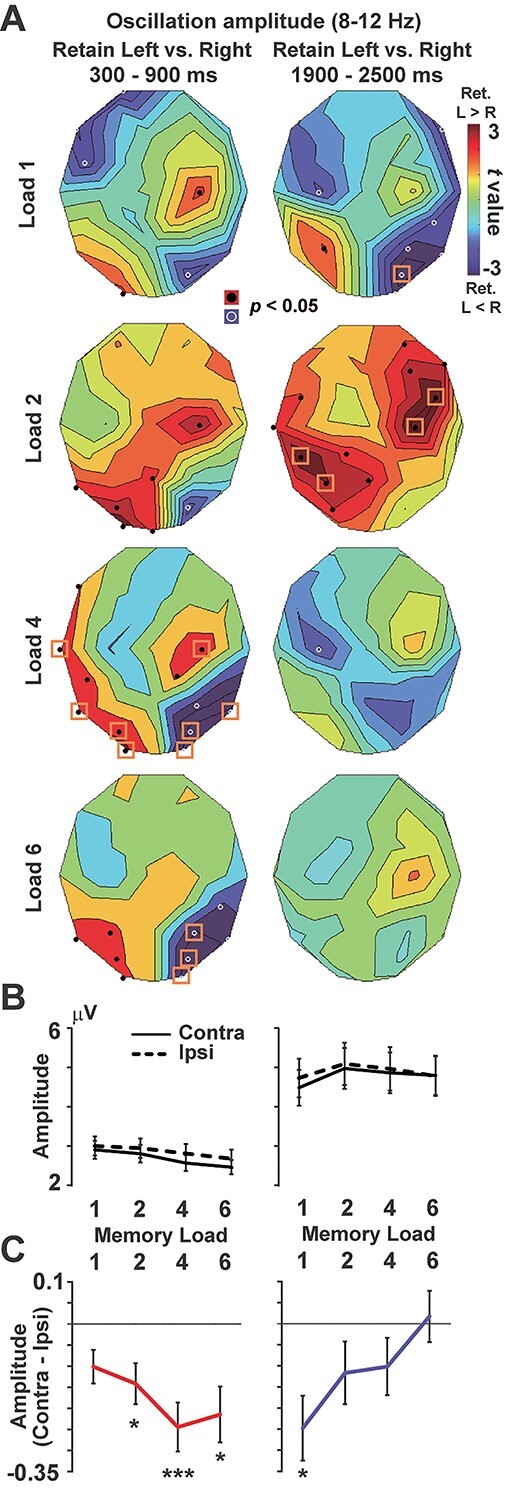
Changes in amplitude of alpha rhythm (8–12 Hz). **A**) *t*-maps, **B**) mean amplitude averaged across the 8 SOIs, **C**) difference between contralateral and ipsilateral conditions. In the early period (300–900 ms, left panels), memory-related responses (reduced amplitudes in the contralateral than ipsilateral conditions) were clearly observed in high-load rather than load–load conditions. In contrast, those signals were more prominent in low-load conditions in the late period (1900–2500 ms, right panels). ^*^P < 0.05, ^*^^*^^*^P < 0.001, 1-group *t*-test corrected with the Bonferroni method. All error bars denote SE across participants.

We then tracked a temporal decrease of vWM signals by contrasting an early (300–900 ms) and late (1900–2500 ms) periods of the retention interval. The definition of the early period (300–900 ms) was based on previous studies (Vogel and Machizawa 2004; Fukuda et al. 2015). Data from 0–300 ms were not used, because they might contain visually evoked responses to the cue and memory array (although it was hard to rule out those sensory responses even in the time window of 300–900 ms). The late period, on the other hand, was defined as an interval of the same length (last 600 ms) of the retention period. As sensors of interest (SOIs), we selected 8 channels over the parieto-occipital region (P3/P4, P7/P8, O1/O2, and PO3/PO4, shown as red in Fig. 2B) based on previous studies (Vogel and Machizawa 2004; Mazaheri and Jensen 2008; Fukuda et al. 2015). Mean ERP amplitudes at 300–900 ms were averaged across the 8 SOIs and shown in left panels of Fig. 3B. Namely, mean ERPs at P3, P7, O1, and PO3 in Retain-Right trials were averaged with those at P4, P8, O2, and PO4 in Retain-Left trials (as the contralateral condition). Differences between the contralateral and ipsilateral conditions (CDA) were shown in Fig. 3C. The same analyses were made for alpha amplitude (Fig. 4).

**Fig. 5:**
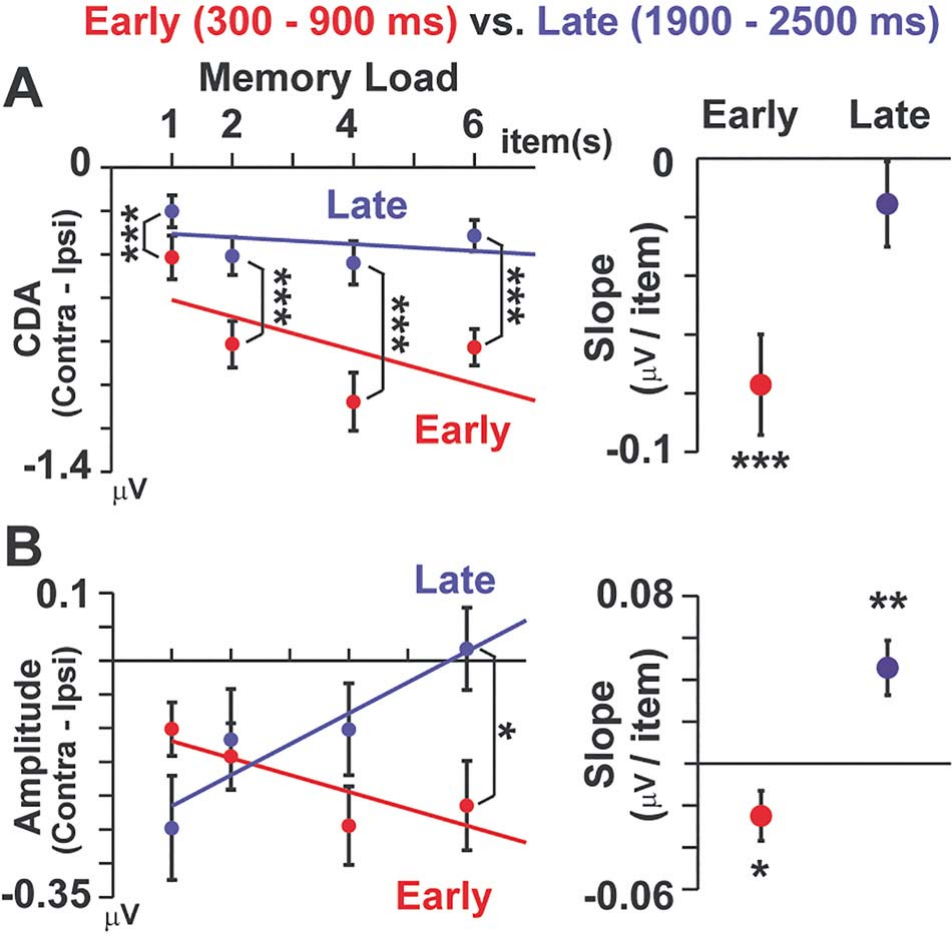
Direct comparisons of memory-related signals between the early versus late periods. **A**) CDA. The data in the left panel are the same as Fig. 3C. Significant reductions of CDA were seen in all loads (left panel), showing the “load-independent” decline from the early to late periods. A slope of linear regression (change in CDA as a function of memory load, right panel) was significantly negative in the early period but not in the late period. **B**) Differential alpha amplitude (contra–ipsi). A significant reduction between the early and late period was selectively seen in load 6, showing the “load-dependent” decline. Slopes of linear regression (right panel) were significantly negative in the early period but positive in the late period. ^*^P < 0.05, ^*^^*^P < 0.01, ^*^^*^^*^P < 0.001, paired *t*-test (early vs. late) in left panels and 1-group *t*-test in right panels. All error bars denote SE across participants.

Finally, we performed a direct comparison of the early and late periods in Fig. 5 (left panels), using a 2-way analysis of variance (ANOVA) of memory loads (1/2/4/6) × retention periods (early/late). For each period, a linear regression line was also computed to evaluate the load effect (changes in vWM signals as an increase in memory load, red, and blue lines). Slopes of those regression lines are shown in the right panels of Fig. 5.

## Results

### Behavioral data (Exp. 1)

Fig. 1B shows changes in task accuracy as a function of a retention interval. Clear decreases in accuracy were observed in load-4 (green line) and load-6 trials (red line). Slopes of linear regression (Fig. 1C) were significantly negative in load 4 (*t*(13) = 4.75, corrected *P* = 0.002, 1-group *t*-tests with the Bonferroni correction) and load 6 (*t*(13) = 4.22, *P* = 0.004) but not in load 1 (*t*(13) = 1.48, uncorrected *P* = 0.16) and load 2 (*t*(13) = 0.81, uncorrected *P* = 0.43). These data successfully replicated the previous findings (e.g. Pertzov et al. 2017) that WM fidelity in high-load conditions rapidly degraded within 2–3 s while that in low-load condition was preserved.

### Behavioral data (Exp. 2)

Means and standard errors (SEs) of hit rates for the change trial were 97.26 ± 0.82% (load 1, an average of L1 and R1), 95.64 ± 0.86% (load 2), 81.73 ± 2.02% (load 4), and 63.98 ± 2.05% (load 6). Mean FA rates for the no-change trial were 1.22 ± 0.33% (load 1), 3.07 ± 0.49% (load 2), 20.34 ± 1.44% (load 4), and 29.53 ± 2.30% (load 6). Memory capacity *K* estimated from those hit and FA rates were 0.96 ± 0.01 (load 1), 1.85 ± 0.02 (load 2), 2.46 ± 0.10 (load 4), and 2.07 ± 0.11 (load 6). These results were consistent with previous studies using the change-detection task (Vogel and Machizawa 2004; Fukuda et al. 2015). Finally, percentage of “change” response in the catch trials was 3.65 ± 0.72%. This was far below the hit rate in load-2 trials (95.64%), showing that the participants correctly moved their attention to a cued hemifield.

### Contralateral delay activity

Fig. 3A shows *t*-maps (Retain Left vs. Retain Right) of ERP for each memory load. In case of the top-left panel, mean ERP amplitude at 300–900 ms in L1 was compared with that in R1. We observed clear CDA over the parieto-occipital cortex in the early (300–900 ms, left panels) and late (1900–2500 ms, right panels) periods of the retention interval. ERP amplitudes averaged across the 8 SOIs are shown in Fig. 3B. Solid and dotted lines denote ERP amplitudes when participants memorized items in a visual field contralateral and ipsilateral to a sensor position, respectively. Differential amplitudes between contralateral and ipsilateral conditions (CDA) are shown in Fig. 3C. One-group *t*-test for each memory load (corrected with the Bonferroni method) indicated significant CDAs at load 1 (*t*(36) = 4.14, corrected *P* < 0.001, Cohen’s *d* = 0.96), load 2 (*t*(36) = 7.58, *P* < 0.001, *d* = 1.76), load 4 (*t*(36) = 8.09, *P* < 0.001, *d* = 1.88), and load 6 (*t*(36) = 9.84, *P* < 0.001, *d* = 2.29) in the early retention period. Significant CDA was also detected in the late period; load 1 (*t*(36) = 2.75, *P* = 0.037, *d* = 0.64), load 2 (*t*(36) = 4.69, *P* < 0.001, *d* = 1.09), load 4 (*t*(36) = 4.41, *P* < 0.001, *d* = 1.03), and load 6 (*t*(36) = 4.37, *P* < 0.001, *d* = 1.02).

### Oscillation amplitude

We next analyzed memory-related changes in alpha amplitude. In the left parieto-occipital cortex, we observed a decrease in oscillation amplitude when participants memorized items in right than left visual field (Retain Left > Retain Right). In the right hemisphere, on the other hand, the decrease in amplitude was seen in Retain Left trials (Retain Left < Retain Right). Differential *t*-maps (Retain Left—Retain Right) thus showed positive and negative *t*-values in the left and right parieto-occipital cortex, respectively (Fig. 4A). In the early period, this contrast between the 2 hemispheres became clearer with an increase in memory load (Fig. 4A, left panels). Differences between contralateral and ipsilateral conditions (Fig. 4C) were significant in load 2 (*t*(36) = 2.90, *P* = 0.025, *d* = 0.68), load 4 (*t*(36) = 4.21, *P* < 0.001, *d* = 0.98), and load 6 (*t*(36) = 3.25, *P* = 0.01, *d* = 0.76). Although those memory-related changes were attenuated in the late period (right panels), we observed a significant difference between contralateral and ipsilateral conditions in load 1 (Fig. 4C, *t*(36) = 3.21, corrected *P* = 0.011, *d* = 0.75), suggesting that the memory-related signal was preserved in the late period when a memory load was low.

As a secondary measure of oscillatory signal, we also analyzed a speed of alpha/beta rhythm during the retention period (Babu Henry Samuel et al. 2018; Noguchi and Kakigi 2020). This analysis provided results consistent with alpha amplitude, showing the memory-related acceleration of brain rhythm in high-load condition of the early period but in low-load conditions of the late period (Fig. S1 in Supplementary Material).

### Direct comparison between the early and late periods

Results of direct comparisons between the early and late periods are shown in left panels of Fig. 5. Red and blue points indicate hemisphere-specific memory responses (contralateral—ipsilateral) in the early and late periods (the same data as Figs. 3C and 4C). In the CDA (upper panel), a 2-way ANOVA of memory load (1/2/4/6) × retention period (early/late) with the Greenhouse–Geisser correction indicated significant main effects of load (*F*(2.33,83.84) = 10.91, *P* < 0.001, *η*^2^ = 0.233) and period (*F*(1,36) = 89.29, *P* < 0.001, *η*^2^ = 0.713) as well as their interaction (*F*(3,108) = 27.29, *P* < 0.001, *η*^2^ = 0.431). A post hoc test between the early and late periods for each memory load revealed a significant reduction of CDA in load 1 (Bonferroni-corrected *P* < 0.001), load 2 (*P* < 0.001), load 4 (*P* < 0.001), and load 6 (*P* < 0.001). In the oscillation amplitude (lower panel), the ANOVA yielded no main effect of load (*F*(3,108) = 2.46, *P* = 0.067, *η*^2^ = 0.064) or period (*F*(1,36) = 1.26, *P* = 0.27, *η*^2^ = 0.034) but showed a significant interaction (*F*(1.8,64.62) = 9.50, *P* < 0.001, *η*^2^ = 0.209). A post hoc test showed a significant difference of early versus late in load 6 (corrected *P* = 0.025).

**Fig. 6:**
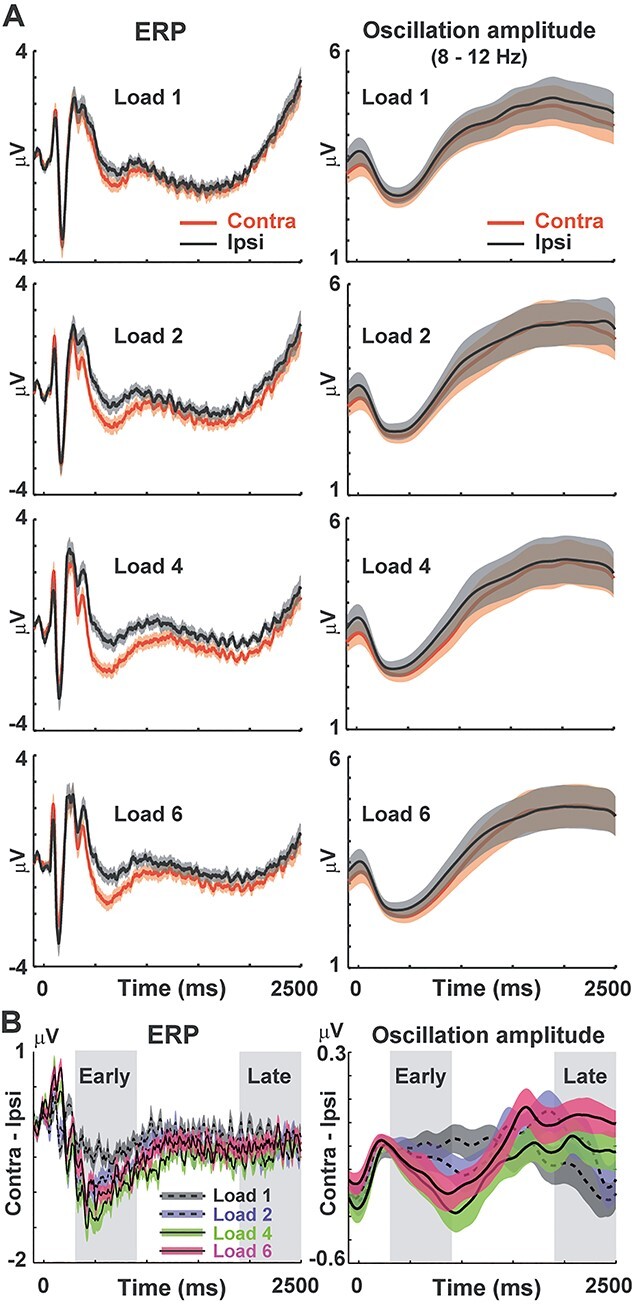
Full-time courses of ERP and alpha amplitude averaged across the 8 SOIs. **A**) Comparisons between contralateral (orange) and ipsilateral (black) conditions in each memory load. Background shadings denote SE across 37 participants. **B**) Differential waveforms (mean ± SE across participants). Note that alpha amplitude (right panel) in the late period showed a reversed load effect in which memory-related responses (decrease in amplitude) was more evident in low-load trials (black and blue) than high-load conditions (green and red).

Results of the linear regression analysis (changes in vWM signals regressed by memory load) are shown in right panels of Fig. 5. In CDA, the slope of a regression line was significantly negative in the early period (*t*(36) = −4.51, *P* < 0.001, *d* = −1.05) but not in the late period(*t*(36) = −1.07, *P* = 0.29, *d* = −0.25), indicating a decline of the load effect over time. In alpha amplitude, the slope was significantly negative in the early period (*t*(36) = −2.10, *P* = 0.043, *d* = −0.49) but significantly positive in the late period (*t*(36) = 3.50, *P* = 0.0012, *d* = 0.81), indicating a reversal of the load effect. These differences between CDA and alpha amplitude were also seen in time courses in Fig. 6.

## Discussion

In the present study, we investigated changes in neural signatures of vWM over time. In the early period of a retention interval (300–900 ms), both CDA and alpha amplitude showed a similar pattern of WM activity that strengthened with a memory load (low loads < high loads). In the late period, the CDA showed a significant temporal decline in all memory loads (Fig. 5A, left). As shown by the significant interaction of memory load (1/2/4/6) × retention period (early/late), this decline was more evident in high-load than low-load trials, which partly resembled the load-dependent decline in behavioral data (Fig. 1C). On the other hand, the direct comparison of alpha amplitude between early versus late (Fig. 5B, left) indicated a selective decline of memory-related signals in load 6. Moreover, the alpha amplitude showed a reversed load effect in the late period; vWM signal was stronger in low-load than high-load conditions (Fig. 5B, right). Taken together, the load-dependent decline (long-term preservation of low-load memory) was more clearly observed in the oscillation amplitude than CDA. These results indicate different roles of CDA and alpha amplitude in a maintenance of vWM contents, consistent with several EEG studies recently published (Hakim et al. 2019; Jin et al. 2020).

Behavioral data have shown that a fidelity of WM was preserved longer as a number of memory item was smaller (Pertzov et al. 2017; Panichello et al. 2019). In contrast, neural underpinnings of this load-dependent decline have remained to be investigated, because most EEG studies on vWM have set a short retention period around 1 s. Although some studies set a longer retention period of > 1 s, they reported a general (load-independent) decrease of memory-related signals over time (Fukuda et al. 2015; Diaz et al. 2021; Li et al. 2021). In this sense, our data of alpha amplitude filled a gap between behavioral and EEG data in previous studies, proposing a neural correlate of a long-term preservation of WM fidelity selective to low-load conditions.

Why was the long-term preservation of WM represented in oscillatory measures? We presume that a key factor associating those 2 is attention. A classical model of cognitive psychology proposed a critical role of attention in converting an iconic memory (which normally degrades within 1 s; Sperling 1960) into a short-term memory (Shiffrin et al. 1974). This role of attention in preserving memory is also supported by recent evidence. For example, Pertzov et al. (2017) showed that a presentation of a retro-cue protected vWM from temporal decline. Participants in their experiment memorized 4 items (bars) with various orientations and reported an orientation of one of the 4 bars randomly determined after a delay (0.1–3 s). When a retro-cue was presented just after the memory array that predicted a color of bar to report later, a fidelity of that item was well preserved even after the longest delay of 3 s. Focusing attention to a subset of items therefore is an effective way to prevent temporal decline. Consistent with this view, changes in alpha amplitude was closely associated with a spatial allocation of attention (Worden et al. 2000; Bauer et al. 2014). The reversed load effect of alpha amplitude in the late period (Fig. 5) thus would reflect attentive processing of 1 or 2 memory item(s), which prevented a temporal degradation and achieved a long-term preservation of WM fidelity.

## Funding

This work was supported by KAKENHI Grants Number 19H04430 from the Japan Society for the Promotion of Science (JSPS) to Y.N.

## Notes

We thank Nahomi Sato, Taeko Kaneda, and Shinobu Maeda for their technical supports. *Conflict of interest statement*: None declared.

## Data availability

All data can be obtained from the corresponding author upon reasonable request.

## Supplementary Material

FigureS1_tgac015

Figure_S1_tgac015
