# Supplementary figures and images for: Neural correlates of a load-dependent decline in visual working memory
Source: Cereb Cortex Commun. 2022 Apr 9;3(2):tgac015. doi: 10.1093/texcom/tgac015 (PMC9050239; doi:10.1093/texcom/tgac015)

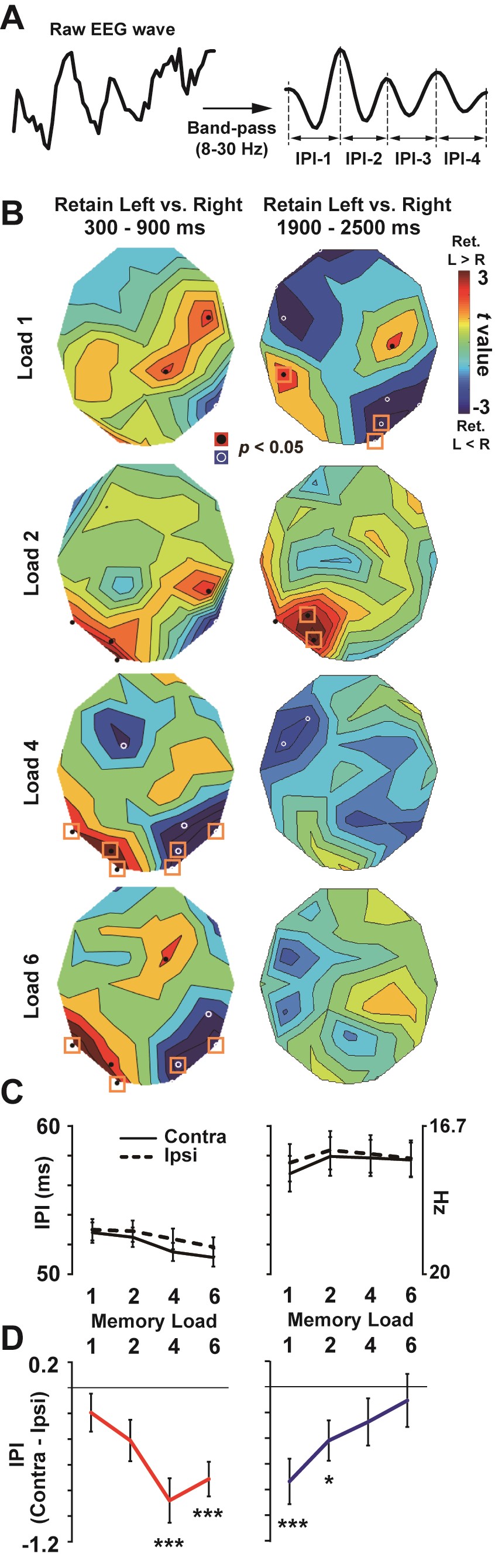

Supplement: FigureS1_tgac015 [file figures1_tgac015.jpeg]
