# Supplementary material for: Neural correlates of a load-dependent decline in visual working memory
Source: Cereb Cortex Commun. 2022 Apr 9;3(2):tgac015. doi: 10.1093/texcom/tgac015 (PMC9050239; doi:10.1093/texcom/tgac015)
Supplement: Figure_S1_tgac015 [file figure_s1_tgac015.pdf]

Figure S1. Memory-related changes in oscillation speed. (A) Procedures. A raw EEG waveform was band-pass filtered (8- 30 Hz). The speed of oscillatory signal was measured as a mean of inter-peak intervals (IPIs) of the filtered waveforms in a given period (e.g. 300 – 900 ms for the early period). A longer mean IPI indicates a slower oscillatory signal. (B) t-maps of mean IPI (Retain Left vs. Retain Right for each memory load) (C) Mean IPI averaged across the 8 SOIs, (D) Difference between contralateral and ipsilateral conditions. Memory-related responses (reduced IPIs in the contralateral than ipsilateral conditions) were clearly seen in high-load conditions in the early period (300 – 900 ms, left panels) but in low-load conditions in the late period (1900 – 2500 ms, right panels). *$p < 0.05$, ***$p < 0.001$, one-group t-test corrected with the Bonferroni method. All error bars denote standard error across participants.
